# Rapid and robust phylotyping of *spa* t003, a dominant MRSA clone in Luxembourg and other European countries

**DOI:** 10.1186/1471-2334-13-339

**Published:** 2013-07-23

**Authors:** David M Engelthaler, Erin Kelley, Elizabeth M Driebe, Jolene Bowers, Carl F Eberhard, Jesse Trujillo, Frederic Decruyenaere, James M Schupp, Joel Mossong, Paul Keim, Jos Even

**Affiliations:** 1 Translational Genomics Research Institute, 3051 W. Shamrell Blvd, 86001, Flagstaff, AZ, USA; 2 Laboratoire National de Santé, Luxembourg, USA; 3 Northern Arizona University, Flagstaff, AZ, USA

**Keywords:** spa t003, MRSA, SNP assays, WGST, Luxembourg, Phylotyping, Molecular epidemiology

## Abstract

**Background:**

*spa* typing is a common genotyping tool for methicillin-resistant *Staphylococcus aureus* (MRSA) in Europe. Given the high prevalence of dominant clones, *spa*-typing is proving to be limited in its ability to distinguish outbreak isolates from background isolates. New molecular tools need to be employed to improve subtyping of dominant local MRSA strains (e.g., *spa* type t003).

**Methods:**

Phylogenetically critical, or canonical, SNPs (can-SNPs) were identified as subtyping targets through sequence analysis of 40 MRSA whole genomes from Luxembourg. Real-time PCR assays were designed around target SNPs and validated using a repository of 240 previously sub-typed and epidemiologically characterized Luxembourg MRSA isolates, including 153 community and hospital isolates, 69 isolates from long term care (LTC) facilities, and 21 prospectively analyzed MRSA isolates. Selected isolates were also analyzed by whole genome SNP typing (WGST) for comparison to the SNP assays and other subtyping techniques.

**Results:**

Fourteen real-time PCR assays were developed and validated, including two assays to determine presence of *spa* t003 or t008. The other twelve assays successfully provided a high degree of resolution within the t003 subtype. WGST analysis of the LTC facility isolates provided greater resolution than other subtyping tools, identifying clusters indicative of ongoing transmission within LTC facilities.

**Conclusions:**

canSNP-based PCR assays are useful for local level MRSA phylotyping, especially in the presence of one or more dominant clones. The assays designed here can be easily adapted for investigating t003 MRSA strains in other regions in Western Europe. WGST provides substantially better resolution than other typing methods.

## Background

The evolution of methicillin resistant *Staphylococcus aureus* (MRSA) is well documented to be heavily influenced by the success of apparently highly fit clones [1]. Local MRSA populations are frequently dominated by such successful clones, which will vary depending on geographic location [2]. These dominant clones typically shape much of the endemic and epidemic spread of this pathogen. This paradigm provides obstacles to performing accurate and informative genotyping for molecular epidemiologic investigations, namely an inability to separate outbreak strains from background strains as well as a inability to examine differences among outbreak strains.

*spa* typing has become a popular genotyping tool in Europe due to its portability and reproducibility [3,4]. *spa* typing is based on the polymorphic direct repeats found in the staphylococcal protein a (*spa*) gene. Differentiation is based on repeat changes due to point mutations, insertions, deletions and duplications [3]. Genotyping with *spa* has shown to have good correlation with multi-locus sequence typing (MLST), both of which have generally acceptable phylogenetic associations, based on population data derived from whole genome sequencing [4]. MLST, *spa*, and other typing methods have therefore provided an excellent basis for understanding the molecular evolution of *S. aureus* and establishing the major clades, or clonal clusters and dominant sub-clades, or clones, in the global population [5-7]. Unfortunately, like MLST, *spa* typing is limited in its resolving power; for instance in a recent survey of *spa* types throughout Europe, five *spa* types accounted for nearly half of all MRSA strains identified [8]. Therefore, these methods are unable to provide sufficient utility for resolving isolates in local epidemic situations [6]. In addition, the negative impact of homoplasies, (e.g., character state conflict resulting from convergent evolution) has been well established in *spa* typing [2,9].

While numerous *spa* types have been seen in Luxembourg, *spa* t003 is clearly the current dominant genotype [10]. *spa* t003 is a highly successful clonal population that was introduced to Europe in the mid 1990s [7] where it has successfully replaced *spa* t008 as the dominant clone in neighboring countries such as Germany [11]. As such, *spa* typing MRSA strains provides limited utility for molecular epidemiology in Luxembourg, constraining health officials’ ability to accurately detect and respond to outbreaks in hospitals and the community. Here we describe efforts to develop improved genotyping for MRSA isolates, particularly within the *spa* t003 clone in Luxembourg, based initially on whole genome sequencing to precisely define the population structure within this narrow clonal complex. Next we identify phylogenetically critical canonical SNPs (canSNPs) for branch-specific PCR-based assays [12] for sensitive and high through put analysis. Note: *spa* t003 is predominantly composed of MLST sequence types ST225 and ST710 [10]; for clarity we refer primarily to *spa* type rather than other genotyping nomenclature, unless otherwise stated.

## Methods

### Selection of strains

Four sets of MRSA strains isolated in Luxembourg were selected from the Laboratoire National de Santé (LNS) strain repository, including an Assay Development Panel, Assay Validation Panel, Long Term Care Facility collection, and a Prospective Hospital isolate collection (see Table 1). The Assay Development Panel included 40 retrospectively collected isolates, including 10 pairs of hospital-, time-, and genotype-linked (by *spa* and MLST) t003 strains; 5 liked t008 pairs; and 10 non-t003 or t008 isolates (Additional file 1: Table S1). The Assay Validation Panel consisted of 153 predominantly *spa* t003 strains from the LNS repository (including community and health care associated strains, Additional file 2: Table S2). The isolates for both of these panels were collected as part of standard of care from Luxembourg hospitals and submitted to the LNS as the national microbiology reference laboratory. The Long Term Care (LTC) Facility collection included 69 previously described strains from 17 long term care facilities in Luxembourg, with nearly half the strains typed as t003 [10] (Additional file 3: Table S3). These isolates were part of a nationally representative cross-sectional MRSA prevalence study in LTCF facilities was conducted from February to June 2010 in the Grand Duchy of Luxembourg [10]. The Prospective Hospital collection included 21 isolates submitted to LNS in March and April of 2012 from three Luxembourg Hospitals (Additional file 4: Table S4). The isolates from the prospective hospital collection were collected as part of standard of care from Luxembourg hospitals and submitted to the LNS as the national microbiology reference laboratory. No patient identifiers were linked to any isolates, all isolates were stored as bacterial isolates and no human subjects were involved in the research.

**Table 1:** MRSA strain sets used for development and validation of PCR assays

**Strain set**	**Isolate no.**	***spa*Types present (no. per type)**
Assay Development	40	t003 (20); t008 (9); t136 (1); t011 (4); t002 (2); t306 (2); t032 (1); t051 (1)
Assay Validation	153	t003 (135); t008 (18);
Long Term Care Facility	69	t003 (33); t032 (3); t045 (3); t899 (3); t105 (2); t002 (2); t010 (2); t3803 (2); t3106 (2)
Prospective Hospital	21	t003 (21)

### DNA extraction

*S. aureus* strains were grown on nutrient agar overnight. Genomic DNA was extracted using a Qiagen DNeasy Blood and Tissue Kit as per manufacturer’s instructions (Qiagen, Valencia, CA), with the addition of lysostaphin (Sigma-Aldrich, St Louis, MO) at 200 ug/mL to the enzymatic lysis buffer and an incubation from 1 to 5 hours.

### Sequencing library preparation/sequencing

Following extraction, adequate DNA presence was verified by visualization via agarose gel electrophoresis. Extracted DNA quantities were normalized to 10 to 15 ng/uL in 200 uL, for final yields of 2 to 3 ug prior to library preparation. DNA samples were prepared for multiplexed, paired-end sequencing on the GAIIx Genome Analyzer (Illumina, Inc, San Diego, CA) following the manufacturers protocol. For each isolate, 1-5 μg dsDNA in 200 μl was sheared and then purified using the QIAquick PCR Purification kit (Qiagen). Enzymatic processing of the DNA followed the guidelines as described in the Illumina protocol, but enzymes for processing were obtained from New England Biolabs (New England Biolabs, Ipswich, MA) and the oligonucleotides and adaptors were obtained from Illumina. After ligation of the adaptors, the DNA was run on a 2% agarose gel for 2 hours, after which a gel slice containing 500-600 bp fragments of each DNA sample was isolated and purified using the QIAquick Gel Extraction kit (Qiagen). Individual libraries were quantified with qPCR on the ABI 7900 HT (Life Technologies Corp., Carlsbad, CA) using the Kapa Library Quantification Kit (Kapa Biosystems, Woburn, MA). Based on the individual library concentrations, equimolar pools of 12 to 24 *S. aureus* libraries were prepared at a concentration of at least 1 nM. To ensure accurate loading onto the flowcell, the same quantification method was used to quantify the final pools. The pooled libraries were sequenced on the Illumina GAIIx using “Genomic DNA sequencing primer V2” for 36 cycles*.* A 100 bp read paired-end run was used for all isolates. An average depth of coverage of 80× was obtained for all samples when raw reads were aligned against a reference. The sequence data has been deposited in a public data base [GenBank: SAMN02203196-SAMN0223319].

### Sequence alignment and SNP detection

Illumina WGS data sets were aligned against the Mu50 and 04-02981 reference genomes [13,14] using the short-read alignment component of the Burrows-Wheeler Aligner (BWA) alignment tool [15]. Reads containing insertions or deletions, and those mapping to multiple locations in the reference were removed from the final alignments. SNP detection was conducted using SolSNP [16], a publically available in-house developed tool. SNPs were excluded if they did not meet a minimum coverage of 10× and if the variant was present in less than 90% of the base calls for that position. Additionally, duplicated regions were identified by a self-comparison of reference genomes using MUMmer version 3.22 and SNPs within these repetitive regions were removed. Phylogenetically informative SNPs, from coding and non-coding regions, were extracted from the alignments also using SolSNP. Finally, loci that were not present in all strains were removed and a matrix containing the remaining orthologous SNPs was generated.

### Phylogeny construction

Phylogenetic trees were developed using the maximum parsimony analysis in MEGA5 [17] with nonparametric bootstrapping using 1000 bootstrap replicates. The first overall tree was constructed, including fifteen closely related publically available whole genome sequences (Additional file 5: Table S5), and all trees were rooted with the most basal taxa or group of taxa in each analysis set. The tree matrix data have been deposited in a public database [Dryad Repository: http://dx.doi.org/10.5061/dryad.73p5d].

### SNP Assay design and validation

Candidate canSNPs (i.e., canonical SNPs) demarcating each of the major phylogenetic branches were identified using the in-house script. Real-Time PCR primers and TaqMan minor groove binding allele specific probes were designed in Primer Express software v3.0 (Life Technologies Corp.) for each of the candidate SNPs (Additional file 6: Table S6). SNP assays were screened across the initial panel of 40 strains that were whole genome sequenced. Real-Time PCR was conducted on ABI 7900 HT (Life Technologies Corp.) using TaqMan Genotyping Master Mix (1×) (Life Technologies Corp.), 900 nM forward and reverse primers, and 200 nM of each probe. After screening across the initial panel, successful assays were selected for further validation and screened across the larger panel of 153 predominantly *spa* t003 strains, the panel of 69 strains from LTC facilities, and the panel of 21 prospectively collected hospital strains.

## Results

### Assay development

**Figure 1:**
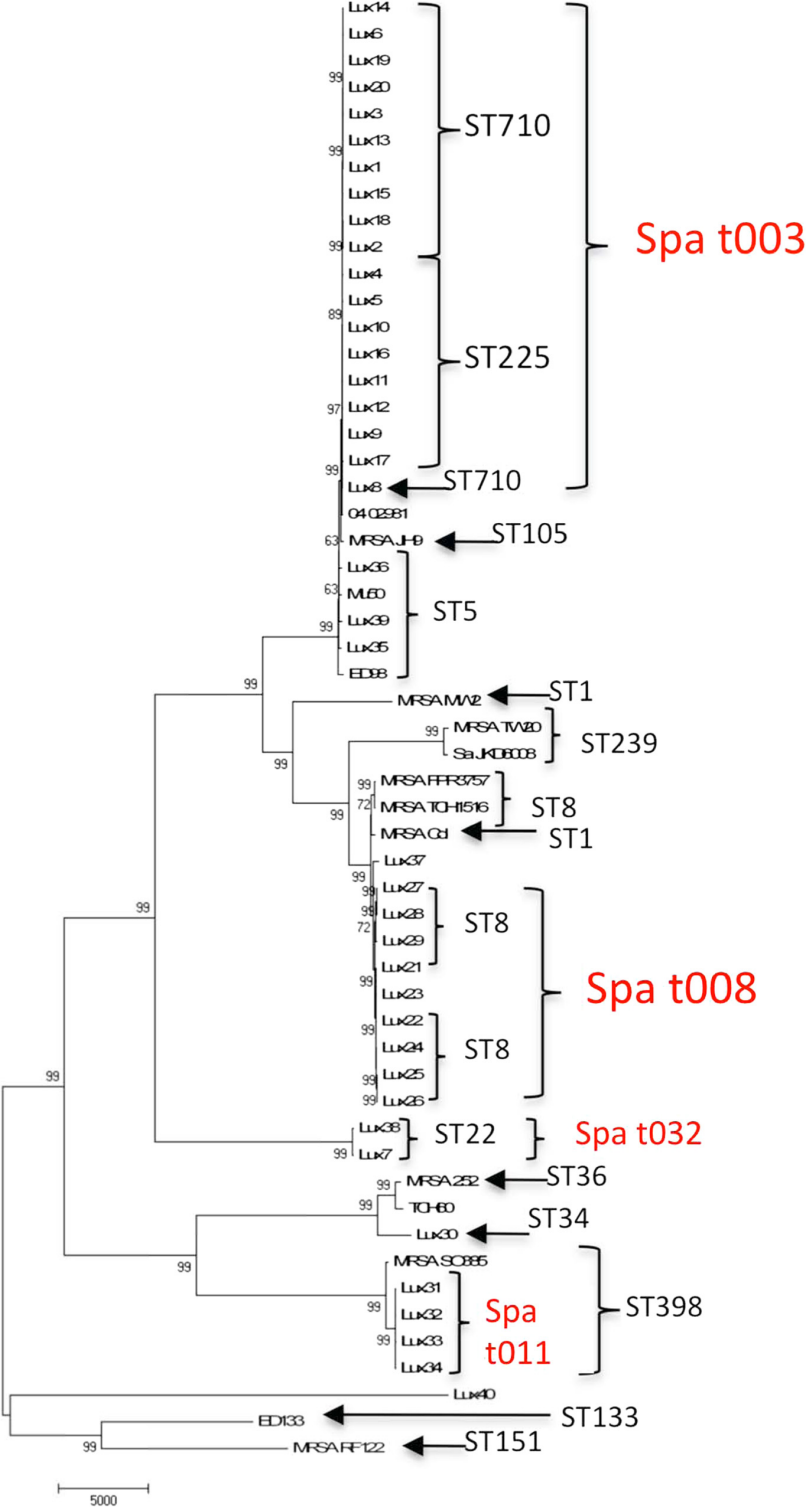
**Whole genome SNP Phylogeny of Assay Development Panel isolates along with fifteen publically available genomes.** The corresponding MLST (black) and *spa* types (red) are noted next to the strains. The phylogenetic relationships are based on a total of 98,768 SNPs out of which 59,284 were parsimony informative with a CI = 0.65. Numbers next to branches are bootstrap values of 100 replicates. Bar length indicates number of SNPs.

**Figure 2:**
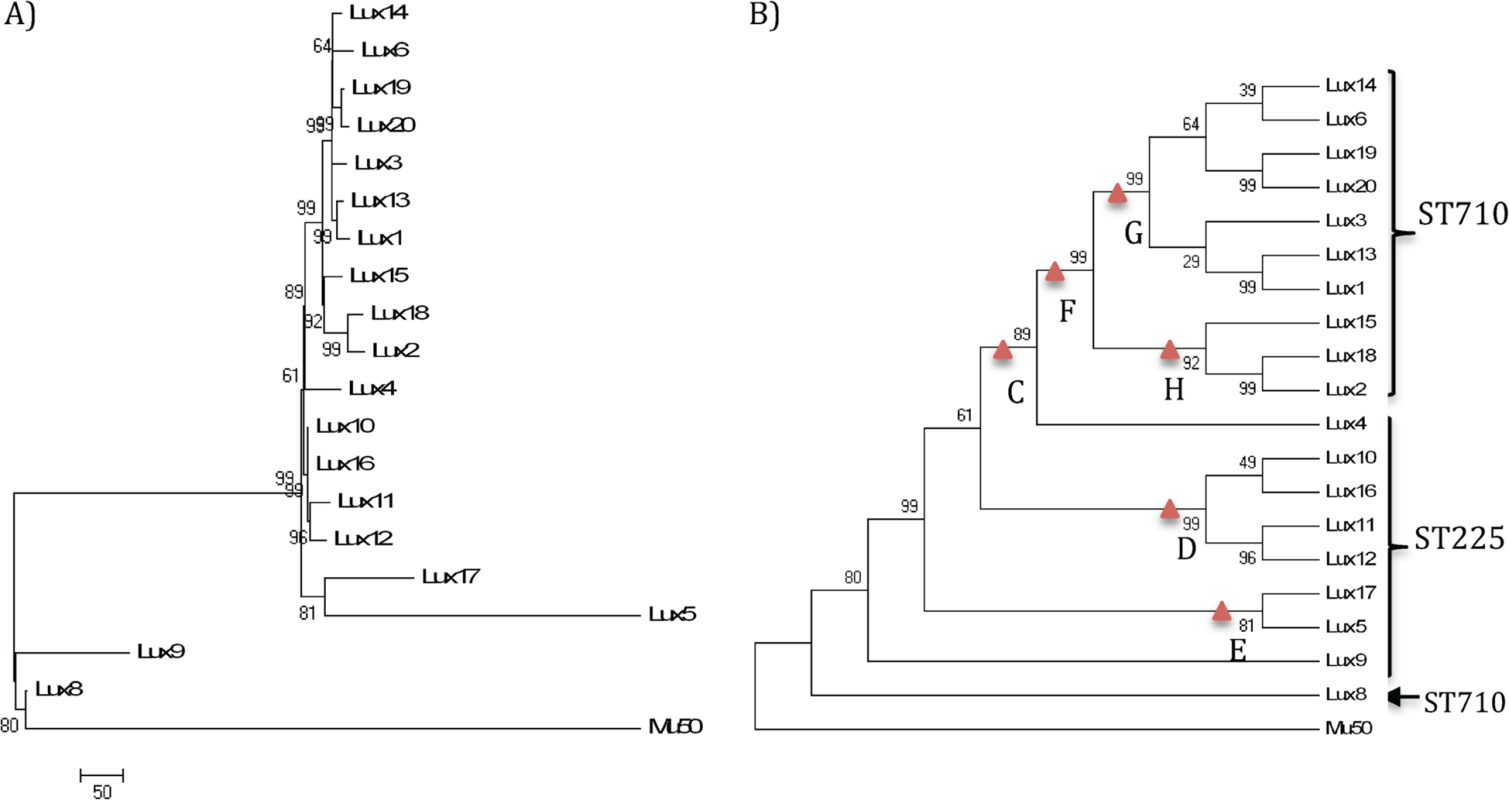
**Phylogenetic (A) and topology (B) trees of Assay Development strains (*spa *t003 only).** Tree includes strains from initial assay development panel. The phylogenetic relationships are based on a total of 1937 SNPs out of which 527 were parsimony informative with a CI = 0.84. Numbers next to branches are bootstrap values of 100 replicates. Branch locations of first six assay SNP targets are indicated with a red triangle.

**Figure 3:**
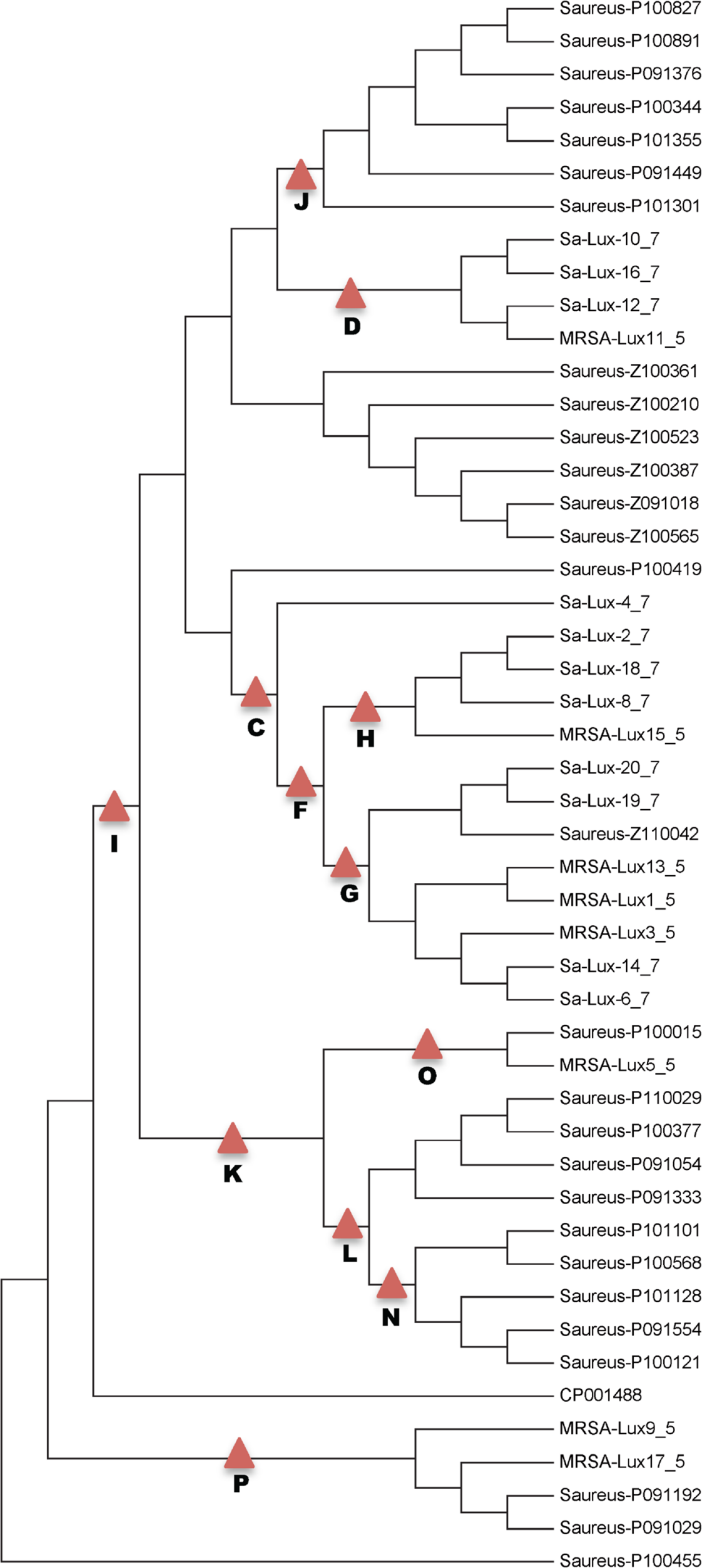
**Final *spa *t003 phylogeny with SNP assay targets.** The phylogenetic relationships are based on a total of 1154 SNPs out of which 193 were parsimony informative with a CI = 0.98. Branch locations of the 12 validated *spa* t003 subtyping assays are indicated with a red triangle. Tree includes 29 additional genomes from Assay Validation Panel to increase resolution within t003.

The whole genome sequence analysis of the Assay Development strain panel provided for a robust phylogenetic tree with well-supported clades and sub-clades, as well as complete discriminations among all strains (Figures 1, and 2). These analyses generally showed phylogenetic concordance with the MLST and spa typing results for these strains (Figure 1). The phylogenetically critical SNPs defining separate clades (i.e., the synapomorphic SNPs found along the branches leading to each clade) provided a candidate canSNP pool for clade-specific assay development. Multiple SNPs were selected from each of six major branches for initial assay development (Figure 2). Validation of the initial six assays with the Validation Panel showed 29 strains (19%) unresolved by the first round of *spa* t003 assays. The 29 unresolved strains were also sequenced and the genomes were added to the *spa* t003 phylogeny to establish improved resolution (Figure 3) - resulting in a total of 12 branches within t003 for which assays 2 were produced.

**Figure 4:**
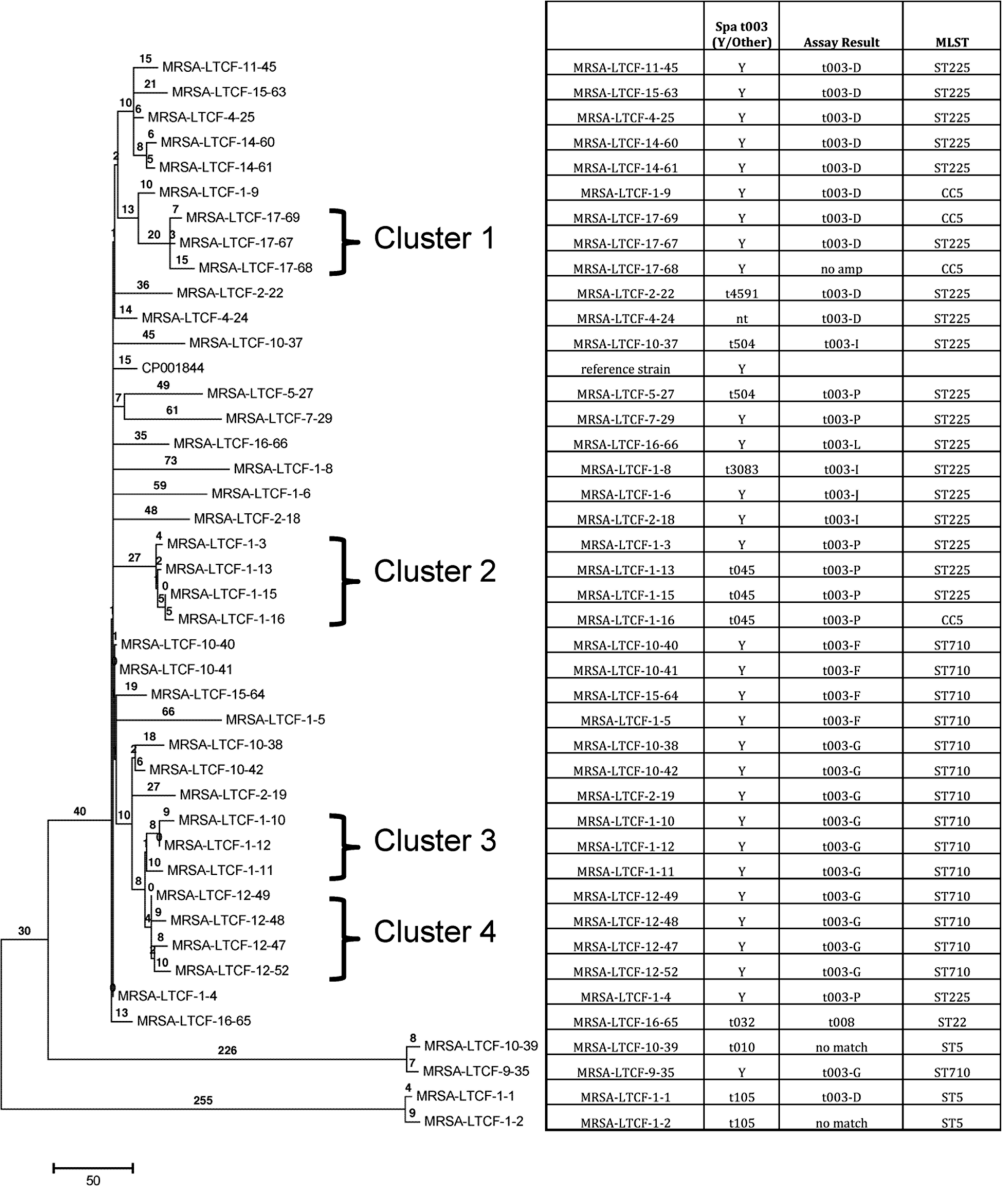
**Whole genome SNP typing (WGST) phylogeny of Long Term Care Facility strains with assay results.** The phylogenetic relationships are based on a total of 1434 SNPs out of which 686 were parsimony informative. The CI is 0.99. Numbers next to branches are numbers of SNPs. The first number in genome name is the facility ID number and the second number is the isolate ID number. Facility linked clusters are highlighted with brackets. Results of the SNP assays in the inset table indicate strain genotypes.

These twelve *spa* t003 subtyping assays, plus assays to distinguish t003 from t008 (see Additional file 7: Figure S1), were screened across the 153 strain Assay Validation panel with greatly improved resolution (Table 2). The canSNP assay results indicate only three (Lux11-12; Lux13-14; Lux19-20) of ten hospital- and genotype-linked t003 isolate pairs from the original Assay Development panel (Additional file 1: Table S1) were actually phylogenetically linked (one of the isolates from the tenth pair would not amplify and therefore was not likely a match to its counterpart isolate). The t003 subtyping assays provided specific identification results for 29 of 30 LTC Facility isolates originally subtyped as t003 (Figure 4), and for all 21 Prospective Hospital isolates (Additional file 4: Table S4).

**Table 2:** Genotyping PCR results from Assay Validation panel

**PCR Results**	**No. Isolates**	**% Isolates**
*spa* t003*	2	1.3%
Branch C	14	9.0%
Branch D	40	25.6%
Branch F	1	0.6%
Branch G	37	23.7%
Branch H	11	7.1%
Branch I	5	3.2%
Branch J	7	4.5%
Branch K	0	0.0%
Branch L	5	3.2%
Branch N	4	2.6%
Branch O	2	1.3%
Branch P	3	1.9%
*spa* t008	18	11.5%
No Match	4	2.6%
Total # samples screened	153	

* no subtype assignment.

The SNP assay analysis and the whole genome SNP typing (WGST) of the LTC Facility collection showed concordant results, with the WGST (Figure 4) providing higher resolution than the more rapid and less expensive canSNP assays, and both showing greater resolution than spa typing and MLST (Additional file 3: Table S3). WGST provided distinct subtypes for each individual strain while establishing phylogenetic relationships between strains. Additionally, WGST identified multiple genomically related clades within in the LTC Facility collection, identifying possible outbreak clusters within different facilities (Figure 4).

## Discussion

We established that canSNP typing, with real-time PCR assays based on phylogeny-defining SNPs, is more informative for subtyping dominant MRSA strains in Luxembourg than other available typing systems. The 12 PCR assays are based on distinct phylogenetic branches identified from the analysis of 69 MRSA whole genomes. These assays distinguished strains that are typically typed as *spa* t003 or t008, as well as provided resolution within t003 (Figure 1). The discriminatory power of these assays was demonstrated by the assignment of specific genotypes for 147 of 149 isolates that had been previously typed only as *spa* type t003. As such, the use of these assays provides both genotype (assignment of genetic similarity) and phylotype (determination of phylogenetic relationships). A recent study [11] identified similar results with the ability to resolve a single distinct sub-clade (named t003-X) within t003 in Germany, using three SNP assays. This particular sub-clade was not identified in the sequenced Luxembourg strains from a bioinformatic analysis of the three SNPs that distinguish that sub-clade (data not shown). As the SNPs identified in this study are presumed to be canonical (branch, clade, or lineage specific) [12], and are useful at resolving numerous clades within t003, it is likely that they will be also useful for genotyping t003 strains in Germany and other European countries with a dominant t003 strain population.

Previous studies have found it necessary to build in numerous assumptions to determine the presence of outbreaks based on *spa* typing. For example, Harmsen [18] used *spa* typing to describe MRSA at a German university hospital for two study periods and determined that a cluster existed if two or more patients with the same *spa* type were seen within 9 days of each other. We confirm the ability to separate hospital-linked strain pairs, using the SNP assays, that were previously indistinguishable by *spa* typing, MLVA, and MLST. Lacking such data, public health and laboratory officials would have possibly assumed direct genetic linkages in the other 12 pairs, and falsely suspecting transmission or shared exposure source. This provides evidence that these SNP-based real-time assays will improve the molecular epidemiologic response to suspect outbreaks of t003 MRSA strains.

With the SNP-based assays, there is still apparent sub-clonal dominance with certain genotypes, particularly the 003-D and 003-G subtypes, which represented 31-52% and 29-37% of t003 stains, respectively, depending on isolate panel (Table 2, Additional file 4: Table S4, Figure 4). Continued monitoring will be needed to determine if this dominance is stable. If it is, then additional SNP assays within these subpopulations can be developed (the canonical SNPs for these groups are available from the existing whole genome data).

Luxembourg samples were dominated by *spa* t003 and t008, as has been described previously [10]. This primary subtype composition indicates possible seeding of the Luxembourg MRSA population from both Germany, which has a dominant *spa* t003, and France, which has a dominant *spa* t008 [8]. Luxembourg is a small country, in area (2,586 km^2^) and population (~520,000), and is situated between France, Germany and Belgium, and has daily commuter, business and visitor travel from these countries. A recent estimate puts the commuter population at approximately 150,000 individuals per day [19]. As such, Luxembourg’s pathogen and commensal ecology is heavily influenced by its neighboring countries. Due to this dominance of t003 and t008, these assays provide distinct utility within Luxembourg. Subtyping methodologies in any region will need to be tied to the local strain populations and may require multiple approaches or a more comprehensive pan-genome approach such whole genome analysis.

The whole genome sequence analysis here provided not only an ability to accurately identify phylogenetically informative SNPs but also the ability to conduct whole genome SNP typing (WGST) to compare to the SNP assays, *spa* typing, MLVA and MLST. WGST has been previously used for advanced molecular typing of MRSA [20,21], as well as for other bacteria [22,23] and fungal pathogens [24-26]. WGST has proven to be far superior to other typing methodologies for determining genetic assignment (genotype) and phylogenetic relatedness (phylotype), given its ability to interrogate genome-wide character states. The WGST and SNP assays closely matched for the Long Term Care Facility isolates, with WGST providing far greater resolution. The identification of multiple clusters of genomically related (i.e., separation by strains by 2 to 10 SNPs) within respective facilities may represent ongoing localized outbreaks within those facilities. These relationships were not apparent by other genotyping analyses.

The phylogeny of all *spa* t003 isolates in the present study, as established by WGST, shows distinct sub-clades, each containing multiple strains, as well as distinct lineages containing solitary strain representatives. This provides evidence of a well-established and diverse clonal structure in Luxembourg. The diversity is possibly fostered through both local evolution of *spa* t003 isolates within Luxembourg as well as continual feeding of *spa* t003 isolates from other t003 dominant countries, such as Germany. WGST-based phylotyping of MRSA t003 strains from predominant commuter-source communities in Germany would be needed to test this hypothesis. Although not studied here, there may be a similar phenomenon with t008 isolates, also possibly having both local evolution and a continual influx of diverse strains from t008 dominant countries (e.g., France and Belgium) [8,27].

## Conclusions

Accurate and resolute genotyping of MRSA (and other bacterial) strains representing dominant clones is necessary to establish true relationships among strains for molecular epidemiological purposes. With the advent of new whole genome exploration tools, we are better able to understand phylogenetic linkage between and among strains. This movement from establishing genotype to establishing phylotype will prove powerful for public health and clinical medicine. SNP-based genotyping assays using real-time PCR are rapid and robust tools for establishing phylotype, as we have shown here. As real-time PCR is a widely accepted methodology in public health and complex clinical laboratories, including at the LNS laboratory in Luxembourg, these assays will advance the capability to resolve *spa* t003 strains. In particular, exclusionary interpretation is possible where an isolate can be shown not to be a member of a particular clade. A lack of membership strongly excludes a recent common evolutionary source, whereas co-membership within a clade is only suggestive. The use of real-time assays provides a significant improvement in time to answer, with results obtained within 2-6 hours (depending on individual laboratory’s sample throughput, preferred extraction methodologies and instrumentation). Per sample costs also vary depending on pooling capabilities and sample throughput, however, costs will generally be less than $25 (USD) per sample. As real-time PCR has become widely implemented, these assays should be readily adaptable by technical staff in any laboratory using this technology.

In the coming years, whole genome sequencing will likely play a primary role in genotyping. Next generation sequencing not only allowed for identification of informative branch-specific markers for rapid genotyping, but also provided further evidence that WGST is the most robust and informative tool for phylotyping strains in outbreak and non-outbreak related scenarios. Currently, this analysis can be done on “tabletop” sequencers (i.e., Illumina MiSeq) within 36 hours for as little as $50 (USD) per sample in sequence reagent costs. As these newer sequencing technologies become even more affordable and portable, and the computational tools become more accessible, it is likely that we will continue to see greater movement towards whole genome analysis for molecular epidemiology and diagnostics of MRSA and other critical pathogens.

## Abbreviations

MRSA: Methicillin resistant *Staphylococcus aureus*; SNP: Single nucleotide polymorphism; canSNP: canonical SNP; PCR: Polymerase chain reaction; LTC: Long term care; WGST: Whole genome SNP typing; MLVA: Multi-locus variable nucleotide tandem repeat analysis; MLST: Multi-locus sequence typing; LNS: Laboratoire National de Santé.

## Competing interests

There are no financial or non-financial competing interests related to this manuscript to declare.

## Authors’ contributions

DE helped conceive study design, assisted with data analysis and was the primary author of the manuscript. EK assisted with laboratory analysis, data analysis and manuscript preparation. ED assisted with study design, laboratory and data analysis, and manuscript preparation. JB assisted with laboratory and data analysis and manuscript review. CE provided the primary bioinformatics analysis of all data. JT conducted the majority of the molecular analysis. FD provided the majority of the microbiology analysis. JS assisted with study design, data analysis and manuscript review. JM assisted with study design and manuscript review. PK assisted with study design and manuscript preparation. JE assisted with study design, laboratory and data analysis, and manuscript preparation. All authors read and approved the final manuscript.

## Authors’ information

David Engelthaler is the former State Epidemiologist for Arizona and is currently the Director of Programs and Operations at TGen North, part of the non-profit Translational Genomics Research Institute. His recent work has focused heavily on the use of next generation sequencing for epidemiologic, diagnostic and forensic purposes.

## Pre-publication history

The pre-publication history for this paper can be accessed here:

http://www.biomedcentral.com/1471-2334/13/339/prepub

## Supplementary Material

Additional file 1: Table S1 Isolate and typing data for Assay Development Panel strains. Click here for file

Additional file 2: Table S2 Isolate and metadata for Assay Validation Panel strains. Click here for file

Additional file 3: Table S3 Isolate and metadata for long term care facility strain panel. Click here for file

Additional file 4: Table S4 SNP Assay Results for Prospective Hospital Panel. Click here for file

Additional file 5: Table S5 Publically available genomes included in phylogeny for Figure 1. Click here for file

Additional file 6: Table S6 Primer and probe sequences for each Real Time PCR Assay. Click here for file

Additional file 7: Figure S1 Phylogenetic (A) and topology (B) trees of initial forty strains. The phylogenetic relationships are based on a total of 13,614 SNPs with a CI = 0.97. Numbers next to branches are bootstrap values of 100 replicates. Branch location of SNP assay targets for identifying *spa* t003 and t008 are indicated with a red triangle. Click here for file
