# Supplementary material for: Rapid and robust phylotyping of spa t003, a dominant MRSA clone in Luxembourg and other European countries
Source: BMC Infect Dis. 2013 Jul 23;13:339. doi: 10.1186/1471-2334-13-339 (PMC3733620; doi:10.1186/1471-2334-13-339)
Supplement: Additional file 1: Table S1 — Isolate and typing data for Assay Development Panel strains. [file 1471-2334-13-339-S1.doc]

Additional files 1: Table S1 Isolate and typing data for Assay Development Panel strains

| **Strain ID** | **Isolate Pair No.** | **Year of collection** | **Hospital** | ***spa* type** | **MLVA Type** | **MLST** | **SNP Assay Result** |
| --- | --- | --- | --- | --- | --- | --- | --- |
| Lux 1 | P1 | 2007 | A | t003 | 130 | 710 | No Amp |
| Lux 2 | P1 | 2007 | A | t003 | 130 | 710 | H |
| Lux 3 | P2 | 2009 | B | t003 | 130 | 710 | F |
| Lux 4 | P2 | 2009 | B | t003 | 130 | 225 | C |
| Lux 5 | P3 | 2009 | B | t003 | 126 | 225 | O |
| Lux 6 | P3 | 2009 | B | t003 | 130 | 710 | F |
| Lux 7 | P4 | 2002 | C | t003 | 130 | 22 | No Match |
| Lux 8 | P4 | 2009 | C | t003 | 130 | 710 | H |
| Lux 9 | P5 | 2004 | D | t003 | 130 | 225 | P |
| Lux 10 | P5 | 2004 | A | t003 | Untypeable | 225 | I |
| Lux 11 | P6 | 2009 | A | t003 | Untypeable | 225 | **I** |
| Lux 12 | P6 | 2009 | A | t003 | Untypeable | 225 | **I** |
| Lux 13 | P7 | 2008 | D | t003 | 130 | 710 | **F** |
| Lux 14 | P7 | 2007 | D | t003 | 130 | 710 | **F** |
| Lux 15 | P8 | 2008 | E | t003 | 130 | 710 | H |
| Lux 16 | P8 | 2008 | E | t003 | 130 | 225 | I |
| Lux 17 | P9 | 2007 | C | t003 | 700 | 225 | P |
| Lux 18 | P9 | 2007 | C | t003 | 130 | 710 | H |
| Lux 19 | P10 | 2007 | F | t003 | 130 | 710 | **F** |
| Lux 20 | P10 | 2007 | F | t003 | 130 | 710 | **F** |
| Lux 21 | P11 | 1997 | B | t008 | 240 | 8 | t008 |
| Lux 22 | P11 | 1997 | B | t008 | 240 | 8 | t008 |
| Lux 23 | P12 | 2002 | C | t008 | 240 | Untypeable | t008 |
| Lux 24 | P12 | 2002 | G | t008 | 240 | 8 | t008 |
| Lux 25 | P13 | 2009 | A | t008 | 240 | 8 | t008 |
| Lux 26 | P13 | 2009 | A | t008 | 240 | 8 | t008 |
| Lux 27 | P14 | 2007 | A | t008 | 314 | Untypeable | t008 |
| Lux 28 | P14 | 2007 | G | t008 | 314 | 8 | t008 |
| Lux 29 | P15 | 2005 | C | t008 | 8 | 8 | t008 |
| Lux 30 | P15 | 2005 | C | t008 | 8 | 34 | No Match |
| Lux 31 | P16 | 2009 | A | t011 | 398 | 398 | No Match |
| Lux 32 | P16 | 2009 | A | t011 | 398 | 398 | No Match |
| Lux 33 | P17 | 2008 | A | t011 | 398 | 398 | No Match |
| Lux 34 | P17 | 2008 | A | t011 | 398 | 398 | No Match |
| Lux 35 | No Pair | 2009 | G | t002 | 91 | 5 | No Match |
| Lux 36 | No Pair | 2009 | A | t002 | 2642 | 5 | No Match |
| Lux 37 | No Pair | 2009 | C | t032 | 22 | Untypeable | No Match |
| Lux 38 | No Pair | 2009 | A | t032 | 22 | 22 | No Match |
| Lux 39 | No Pair | 2009 | B | t0306 | Untypeable | 5 | No Match |
| Lux 40 | No Pair | 2009 | G | t0356 | 444 | Untypeable | No Match |
