# Supplementary material for: Rapid and robust phylotyping of spa t003, a dominant MRSA clone in Luxembourg and other European countries
Source: BMC Infect Dis. 2013 Jul 23;13:339. doi: 10.1186/1471-2334-13-339 (PMC3733620; doi:10.1186/1471-2334-13-339)
Supplement: Additional file 2: Table S2 — Isolate and metadata for Assay Validation Panel strains. [file 1471-2334-13-339-S2.doc]

Additional file 2: Table S2 Isolate and metadata for Assay Validation Panel strains

| **Strain ID** | **Year of collection** | **Hospital** | ***spa* type** |
| --- | --- | --- | --- |
| P090853 | 2009 | 3 | t003 |
| P090872 | 2009 | 3 | t003 |
| P090912 | 2009 | 3 | t003 |
| P090965 | 2009 | 1 | t003 |
| P090977 | 2009 | 1 | t003 |
| P090979 | 2009 | 1 | t003 |
| P090991 | 2009 | 1 | t003 |
| P091029 | 2009 | 3 | t003 |
| P091054 | 2009 | 3 | t003 |
| P091053 | 2009 | 1 | t003 |
| P091065 | 2009 | 1 | t003 |
| P091086 | 2009 | 3 | t003 |
| P091115 | 2009 | 1 | t003 |
| P091127 | 2009 | 3 | t003 |
| Z090791 | 2009 | 6 | t003 |
| P091192 | 2009 | 1 | t003 |
| P091239 | 2009 | 1 | t003 |
| P091301 | 2009 | 3 | t003 |
| P091333 | 2009 | 3 | t003 |
| Z090884 | 2009 | 6 | t003 |
| P091357 | 2009 | 2 | t003 |
| P091368 | 2009 | 1 | t003 |
| P091384 | 2009 | 2 | t003 |
| P091376 | 2009 | 1 | t003 |
| P091425 | 2009 | 2 | t003 |
| P091449 | 2009 | 1 | t003 |
| P091462 | 2009 | 3 | t003 |
| P091554 | 2009 | 3 | t003 |
| Z090997 | 2009 | 6 | t003 |
| P091592 | 2009 | 2 | t003 |
| Z091018 | 2009 | 6 | t003 |
| Z091032 | 2009 | 5 | t003 |
| Z091039 | 2009 | 5 | t003 |
| Z091045 | 2009 | 6 | t003 |
| P100015 | 2010 | 2 | t003 |
| P100011 | 2010 | 2 | t003 |
| P100043 | 2010 | 3 | t003 |
| P100067 | 2010 | 2 | t003 |
| P100082 | 2010 | 1 | t003 |
| P100121 | 2010 | 3 | t003 |
| P100167 | 2010 | 1 | t003 |
| P100175 | 2010 | 2 | t003 |
| P100173 | 2010 | 3 | t003 |
| Z100112 | 2010 | 6 | t003 |
| P100206 | 2010 | 3 | t003 |
| P100252 | 2010 | 3 | t003 |
| P100255 | 2010 | 1 | t003 |
| P100266 | 2010 | 1 | t003 |
| Z100163 | 2010 | 6 | t003 |
| P100320 | 2010 | 3 | t003 |
| P100331 | 2010 | 1 | t003 |
| P100344 | 2010 | 3 | t003 |
| P100354 | 2010 | 4 | t003 |
| P100355 | 2010 | 1 | t003 |
| P100371 | 2010 | 1 | t003 |
| Z100210 | 2010 | 6 | t003 |
| P100377 | 2010 | 3 | t003 |
| P100393 | 2010 | 2 | t003 |
| P100409 | 2010 | 3 | t003 |
| P100419 | 2010 | 1 | t003 |
| P100414 | 2010 | 3 | t003 |
| P100455 | 2010 | 1 | t003 |
| P100490 | 2010 | 3 | t003 |
| P100505 | 2010 | 1 | t003 |
| P100522 | 2010 | 3 | t003 |
| P100529 | 2010 | 2 | t003 |
| P100552 | 2010 | 3 | t003 |
| P100568 | 2010 | 1 | t003 |
| P100619 | 2010 | 2 | t003 |
| P100666 | 2010 | 3 | t003 |
| Z100324 | 2010 | 6 | t003 |
| P100730 | 2010 | 1 | t003 |
| P100736 | 2010 | 3 | t003 |
| P100764 | 2010 | 3 | t003 |
| P100786 | 2010 | 3 | t003 |
| P100793 | 2010 | 1 | t003 |
| P100791 | 2010 | 1 | t003 |
| Z100361 | 2010 | 6 | t003 |
| P100827 | 2010 | 2 | t003 |
| P100891 | 2010 | 1 | t003 |
| P100913 | 2010 | 1 | t003 |
| P100948 | 2010 | 2 | t003 |
| Z100387 | 2010 | 6 | t003 |
| Z100394 | 2010 | 6 | t003 |
| Z100393 | 2010 | 6 | t003 |
| P100991 | 2010 | 2 | t003 |
| P101034 | 2010 | 3 | t003 |
| P101042 | 2010 | 1 | t003 |
| P101076 | 2010 | 2 | t003 |
| P101079 | 2010 | 1 | t003 |
| Z100472 | 2010 | 6 | t003 |
| P101095 | 2010 | 3 | t003 |
| P101101 | 2010 | 3 | t003 |
| Z100476 | 2010 | 6 | t003 |
| Z100480 | 2010 | 6 | t003 |
| P101128 | 2010 | 3 | t003 |
| P101136 | 2010 | 3 | t003 |
| P101164 | 2010 | 2 | t003 |
| P101205 | 2010 | 1 | t003 |
| P101265 | 2010 | 1 | t003 |
| P101287 | 2010 | 1 | t003 |
| P101288 | 2010 | 3 | t003 |
| P101301 | 2010 | 1 | t003 |
| P101317 | 2010 | 2 | t003 |
| Z100523 | 2010 | 6 | t003 |
| P101329 | 2010 | 1 | t003 |
| Z100538 | 2010 | 6 | t003 |
| P101347 | 2010 | 1 | t003 |
| P101355 | 2010 | 1 | t003 |
| P101362 | 2010 | 2 | t003 |
| P101374 | 2010 | 1 | t003 |
| P101410 | 2010 | 1 | t003 |
| P101411 | 2010 | 3 | t003 |
| P101444 | 2010 | 1 | t003 |
| P101459 | 2010 | 2 | t003 |
| P101460 | 2010 | 2 | t003 |
| Z100565 | 2010 | 6 | t003 |
| P101487 | 2010 | 3 | t003 |
| P101485 | 2010 | 1 | t003 |
| P101493 | 2010 | 1 | t003 |
| P101499 | 2010 | 1 | t003 |
| P101508 | 2010 | 1 | t003 |
| P101537 | 2010 | 3 | t003 |
| P101547 | 2010 | 3 | t003 |
| P110004 | 2010 | 1 | t003 |
| P110029 | 2011 | 2 | t003 |
| P110068 | 2011 | 3 | t003 |
| P110071 | 2011 | 1 | t003 |
| P110085 | 2011 | 3 | t003 |
| P110100 | 2011 | 1 | t003 |
| P110140 | 2011 | 1 | t003 |
| P110126 | 2011 | 2 | t003 |
| P110149 | 2011 | 3 | t003 |
| P110167 | 2011 | 3 | t003 |
| P110175 | 2011 | 2 | t003 |
| P110170 | 2011 | 1 | t003 |
| P110240 | 2011 | 1 | t003 |
| P110249 | 2011 | 1 | t003 |
| Z110042 | 2011 | 6 | t003 |
| P110274 | 2011 | 1 | t003 |
| P110278 | 2011 | 1 | t003 |
| P110306 | 2011 | 3 | t003 |
| P110313 | 2011 | 1 | t003 |
| P110315 | 2011 | 3 | t003 |
| P110341 | 2011 | 2 | t003 |
| P110331 | 2011 | 1 | t003 |
| P110346 | 2011 | 2 | t003 |
| P110350 | 2011 | 3 | t003 |
| P110356 | 2011 | 1 | t003 |
| Z110061 | 2011 | 6 | t003 |
