# Supplementary material for: Rapid and robust phylotyping of spa t003, a dominant MRSA clone in Luxembourg and other European countries
Source: BMC Infect Dis. 2013 Jul 23;13:339. doi: 10.1186/1471-2334-13-339 (PMC3733620; doi:10.1186/1471-2334-13-339)
Supplement: Additional file 3: Table S3 — Isolate and metadata for long term care facility strain panel. [file 1471-2334-13-339-S3.doc]

Additional file 3: Table S3 Isolate and metadata for long term care facility strain panel

| **Strain ID** | **Year of collection** | **LTCF ID No.** | ***spa* type** | **MLST** | **Clonal Complex** |
| --- | --- | --- | --- | --- | --- |
| LTCF-1-1 | 2010 | 1 | t105 | ST 5 | 5 |
| LTCF-1-2 | 2010 | 1 | t105 | ST 5 | 5 |
| LTCF-1-3 | 2010 | 1 | t003 | ST 225 | 5 |
| LTCF-1-4 | 2010 | 1 | t003 | ST 225 | 5 |
| LTCF-1-5 | 2010 | 1 | t003 | ST 710 | 5 |
| LTCF-1-6 | 2010 | 1 | t003 | ST 225 | 5 |
| LTCF-1-7 | 2010 | 1 | t3083 | ST 225 | 5 |
| LTCF-1-8 | 2010 | 1 | t3083 | ST 225 | 5 |
| LTCF-1-9 | 2010 | 1 | t003 | ST 225 | 5 |
| LTCF-1-10 | 2010 | 1 | t003 | ST 710 | 5 |
| LTCF-1-11 | 2010 | 1 | t003 | ST 710 | 5 |
| LTCF-1-12 | 2010 | 1 | t003 | ST 710 | 5 |
| LTCF-1-13 | 2010 | 1 | t045 | ST 225 | 5 |
| LTCF-1-14 | 2010 | 1 | t003 | Untypeable | 5 |
| LTCF-1-15 | 2010 | 1 | t045 | ST 710 | 5 |
| LTCF-1-16 | 2010 | 1 | t045 | ST 225 | 5 |
| LTCF-1-17 | 2010 | 1 | t740 | ST 45 | 45 |
| LTCF-2-18 | 2010 | 2 | t003 | ST 225 | 5 |
| LTCF-2-19 | 2010 | 2 | t003 | ST 710 | 5 |
| LTCF-2-20 | 2010 | 2 | t003 | Untypeable | 5 |
| LTCF-2-21 | 2010 | 2 | t003 | ST 710 | 5 |
| LTCF-2-22 | 2010 | 2 | t4591 | ST 225 | 5 |
| LTCF-3-23 | 2010 | 3 | t032 | ST 22 | 22 |
| LTCF-4-24 | 2010 | 4 | nt | ST 225 | 5 |
| LTCF-4-25 | 2010 | 4 | t003 | ST 225 | 5 |
| LTCF-4-26 | 2010 | 4 | t003 | Untypeable | 5 |
| LTCF-5-27 | 2010 | 5 | t504 | ST 225 | 5 |
| LTCF-6-28 | 2010 | 6 | t008 | ST 8 | 8 |
| LTCF-7-29 | 2010 | 7 | t003 | ST 225 | 5 |
| LTCF-7-30 | 2010 | 7 | t899 | ST 398 | 398 |
| LTCF-8-31 | 2010 | 8 | t2849 | ST 8 | 8 |
| LTCF-9-32 | 2010 | 9 | t2054 | ST 8 | 8 |
| LTCF-9-33 | 2010 | 9 | t008 | ST 8 | 8 |
| LTCF-9-34 | 2010 | 9 | t008 | Untypeable | 8 |
| LTCF-9-35 | 2010 | 9 | t003 | ST 710 | 5 |
| LTCF-10-36 | 2010 | 10 | t010 | ST 5 | 5 |
| LTCF-10-37 | 2010 | 10 | t504 | ST 225 | 5 |
| LTCF-10-38 | 2010 | 10 | t003 | ST 710 | 5 |
| LTCF-10-39 | 2010 | 10 | t010 | ST 5 | 5 |
| LTCF-10-40 | 2010 | 10 | t003 | ST 710 | 5 |
| LTCF-10-41 | 2010 | 10 | t003 | ST 710 | 5 |
| LTCF-10-42 | 2010 | 10 | t003 | ST 710 | 5 |
| LTCF-10-43 | 2010 | 10 | t002 | ST 5 | 5 |
| LTCF-11-44 | 2010 | 11 | t216 | ST 59 | 59 |
| LTCF-11-45 | 2010 | 11 | t003 | ST 225 | 5 |
| LTCF-12-46 | 2010 | 12 | t899 |  | 398 |
| LTCF-12-47 | 2010 | 12 | t003 | ST 710 | 5 |
| LTCF-12-48 | 2010 | 12 | t003 | ST 710 | 5 |
| LTCF-12-49 | 2010 | 12 | t003 | ST 710 | 5 |
| LTCF-12-50 | 2010 | 12 | t003 | Untypeable | 5 |
| LTCF-12-51 | 2010 | 12 | t003 | Untypeable | 5 |
| LTCF-12-52 | 2010 | 12 | t003 | ST 710 | 5 |
| LTCF-12-53 | 2010 | 12 | t899 | Untypeable | 398 |
| LTCF-12-54 | 2010 | 12 | t003 | ST 710 | 5 |
| LTCF-12-55 | 2010 | 12 | t3106 | ST 398 | 398 |
| LTCF-12-56 | 2010 | 12 | t003 | Untypeable | 5 |
| LTCF-12-57 | 2010 | 12 | t3106 | Untypeable | 398 |
| LTCF-13-58 | 2010 | 13 | t002 | Untypeable | 5 |
| LTCF-14-59 | 2010 | 14 | t476 | ST 22 | 22 |
| LTCF-14-60 | 2010 | 14 | t003 | ST 225 | 5 |
| LTCF-14-61 | 2010 | 14 | t003 | ST 225 | 5 |
| LTCF-15-62 | 2010 | 15 | t032 | Untypeable | 22 |
| LTCF-15-63 | 2010 | 15 | t003 | ST 225 | 5 |
| LTCF-15-64 | 2010 | 15 | t003 | ST 710 | 5 |
| LTCF-16-65 | 2010 | 16 | t032 | ST 22 | 22 |
| LTCF-16-66 | 2010 | 16 | t003 | ST 225 | 5 |
| LTCF-17-67 | 2010 | 17 | t003 | ST 225 | 5 |
| LTCF-17-68 | 2010 | 17 | t003 | ST 225 | 5 |
| LTCF-17-69 | 2010 | 17 | t003 | ST 225 | 5 |
