# Supplementary material for: Rapid and robust phylotyping of spa t003, a dominant MRSA clone in Luxembourg and other European countries
Source: BMC Infect Dis. 2013 Jul 23;13:339. doi: 10.1186/1471-2334-13-339 (PMC3733620; doi:10.1186/1471-2334-13-339)
Supplement: Additional file 4: Table S4 — SNP Assay Results for Prospective Hospital Panel. [file 1471-2334-13-339-S4.doc]

Additional file 4: Table S4 SNP Assay Results for Prospective Hospital Panel

| **Sample ID** | **Assay Result** | **Facility** |
| --- | --- | --- |
| 1 | 003-D | A |
| 4 | 003-D | A |
| 7 | 003-D | A |
| 10 | 003-D | A |
| 13 | 003-G | A |
| 16 | 003-G | A |
| 19 | 003-G | A |
| 11 | 003-D | B |
| 14 | 003-D | B |
| 20 | 003-D | B |
| 2 | 003-G | B |
| 5 | 003-G | B |
| 17 | 003-G | B |
| 8 | 003-F | B |
| 12 | 003-D | C |
| 15 | 003-D | C |
| 18 | 003-D | C |
| 21 | 003-D | C |
| 9 | 003-G | C |
| 3 | 003-I | C |
| 6 | 003-I | C |
