# Supplementary material for: Rapid and robust phylotyping of spa t003, a dominant MRSA clone in Luxembourg and other European countries
Source: BMC Infect Dis. 2013 Jul 23;13:339. doi: 10.1186/1471-2334-13-339 (PMC3733620; doi:10.1186/1471-2334-13-339)
Supplement: Additional file 5: Table S5 — Publically available genomes included in phylogeny for Figure 1. [file 1471-2334-13-339-S5.doc]

Additional file 5: Table S5 Publically available genomes included in phylogeny for Figure 1

| **Strain ID** | **NCBI Accession #** | **Year of Collection** | **Location** | ***spa* type** | **MLST** | **Clonal Complex** |
| --- | --- | --- | --- | --- | --- | --- |
| 04 02981 | CP001844 | 2004 | Germany | t003 | ST225 | CC5 |
| MRSA JH9 | CP000703 | 2000 | Maryland | t002 | ST105 | CC5 |
| Mu50- Ref | BA000017 | 1996 | Japan | t002 | ST5 | CC5 |
| ED98 | CP001781 | 1996-97 | Ireland | t002 | ST5 | CC5 |
| MRSA MW2 | BA000033 | 1998 | North Dakota | t128 | ST1 | CC1 |
| MRSA TW20 | FN433596 | 2003 | London | t037 | ST239 | CC8 |
| SaJKD6008 | CP002120 | 2006 | New Zealand | t037 | ST239 | CC8 |
| MRSA FPR3757 | CP000255 | 2002 | California | t008 | ST8 | CC8 |
| TCH1516 | CP000730 | 2001 | Texas | t622 | ST8 | CC8 |
| MRSA COL | CP000046 | 1960's (early) | England | t008 | ST250 | CC8 |
| MRSA 252 | BX571856 | 1997 | UK | t018 | ST36 | CC30 |
| TCH60 | CP002110 | unknown | Texas | - | - | - |
| MRSA SO385 | AM990992 | 2003 | Netherlands | t011 | ST398 | CC398 |
| ED133 | CP001996 | 1997 | France | t2678 | ST133 | CC133 |
| MRSA RF122 | AJ938182 | 1993 | Ireland | t529 | ST151 | CC705 |
