# Supplementary material for: Rapid and robust phylotyping of spa t003, a dominant MRSA clone in Luxembourg and other European countries
Source: BMC Infect Dis. 2013 Jul 23;13:339. doi: 10.1186/1471-2334-13-339 (PMC3733620; doi:10.1186/1471-2334-13-339)
Supplement: Additional file 6: Table S6 — Primer and probe sequences for each Real Time PCR Assay. [file 1471-2334-13-339-S6.doc]

Additional file 6: Table S6 Primer and probe sequences for each Real Time PCR Assay.

| **Branch** | **Primer/Probe name** | **Primer/Probe sequence** |
| --- | --- | --- |
| t008 | t008_F_b | AGCAATCGCTATTAAATCAACACAAC |
|  | t008_R_b | CTTGTCTTATAGTCTTGATTGCCTTGTAA |
|  | t008_prb_b_fam | AACGATGAAGCAATTAA |
|  | t008_prb_b_vic | AAACGATGAAGCAGT T |
| t003 | t003_F_b | AGCTACCTGCATTGCCAATTCT |
|  | t003_R_b | GCATTCGGTATTCCTTTAATTGAGA |
|  | t003_prb_b_fam | AACGATTGAACTCCT |
|  | t003_prb_b_vic | AACGATTGAACCCCTT |
| C | branchC2_F | TTGCTTTTTTGATACGTTTCTGTTCT |
|  | branchC2_R | TGTTAAAAGCCAAAAGTGTCAATGA |
|  | branchC2_prb_fam | TACCGCAAACAATATA |
|  | nonbC2_prb_vic | ATATACCGCAAACAACAT |
| D | branchD_F_c | GGTGAATGGTACGAAAAAATT |
|  | branchD_R_c | AAAGCTGTTTCTACTGCTTCA |
|  | branchD_prb_c_fam | CAGGTAAAGATAAAACTT |
|  | branchD_prb_c_vic | CAGGTAAAGATAAAGCTT |
| F | branchF2_F | TTGTCCATACTTGTCCAGGATATAGTTT |
|  | branchF2_R | GAATCCAGCGTCTATGACTAAATTAATG |
|  | branchF2_prb_fam | CTGTGTCATCAAGTAAA |
|  | nonbF2_prb_vic | CTGTGTCATCAAGTGAA |
| G | branchG_F_b | CGCCATTGTCAAACAACGTATT |
|  | branchG_R_b | CGGTTTAGATATTGCAGTGTCTGTAATT |
|  | branchG_prb_b_fam | CGTTTCTTCGGGTACT |
|  | branchG_prb_b_vic | AATAAGGCGTTTCTTCAGGTA |
| H | branchH_F_a | CCATATTACAAATGTCCTAGTGAAATGAT |
|  | branchH_R_a | GTCAAAAAGACTTTGTAATAATGCGTTC |
|  | branchH_prb_a_fam | TTGTACTTTAGAGTTATTGAA |
|  | branchH_prb_a_vic | TTGTACTTTAGAGTTATTAAA |
| I | branchI_F | TGCAATACAAGTATCCATATCAACGA |
|  | branchI_R | CTCACTCTGTAAAACGGATAATTTGAA |
|  | branchI_prb_fam | CAATGTTTTCGCCTCC |
|  | nonBI_prb_vic | ATTTTGCCAATGTCTT |
| J | branchJ_F | ACGCAGATAGCGGTGTGAATT |
|  | branchJ_R | GGCGATTATCTATTCATCAGGTGAT |
|  | branchJ_prb_fam | TTGAATCATATTAACAAGGC |
|  | nonbJ_prb_vic | TTGAATCACATTAACAAGG |
| K | branchK_F | TGTCATCACTGTAGCAACTGGAATATT |
|  | branchK_R | CAGCCGCCCAGATAAACTAGA |
|  | branchK_prb_fam | TGTTAAATTGTTCAGTTAGTACC |
|  | nonbK_prb_vic | TGTTAAATTGCTCAGTTAGT |
| L | branchL_F | CGATACGATCAGCACCTAAATCTG |
|  | branchL_R | CGTCTAATTCAAGAACTTGCACATG |
|  | branchL_prb_fam | ATGATCTTGCCTTTCATGTGTA |
|  | nonbL_prb_vic | TGTGGATGATCTTGTCT |
| N | branchN_F | CAATAGAATTAATTGGAGGATTCACG |
|  | branchN_R | GAACACTTGTTTCATGCAGACAAA |
|  | branchN_prb_fam | CGCGATTGAGGGCGA |
|  | nonbN_prb_vic | CGATTGAGGGTGAAT |
| O | branchO_F | TGGCTGGATTAATGAGCTTTTTG |
|  | branchO_R | CGGCCAGTTGTTGTAGAACGT |
|  | branchO_prb_fam | AGGCTTCAATATTTCCAT |
|  | nonbO_prb_vic | CTTTTTATGAGGCTTCAACAT |
| P | branchP_F | TTTGGCGGTGAAGGTGTTG |
|  | branchP_R | AAATAACTTGGTGTTTTCCCAATCA |
|  | branchP_prb_fam | TTGATTTATGTCATGTTAAC |
|  | nonbP_prb_vic | TGATTTATGTCGTGTTAACG |
