# Supplementary figures and images for: Rapid and robust phylotyping of spa t003, a dominant MRSA clone in Luxembourg and other European countries
Source: BMC Infect Dis. 2013 Jul 23;13:339. doi: 10.1186/1471-2334-13-339 (PMC3733620; doi:10.1186/1471-2334-13-339)

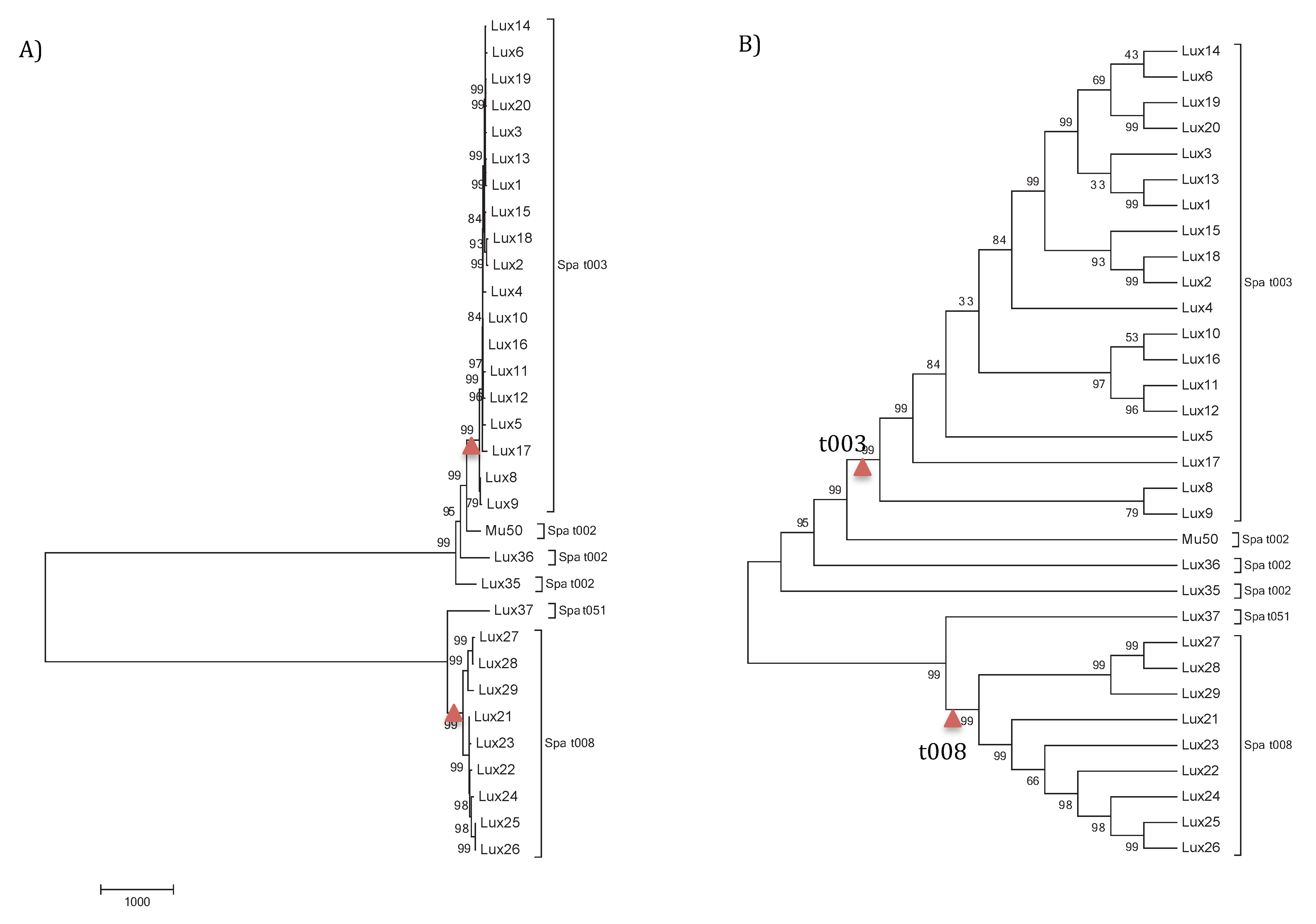

Supplement: Additional file 7: Figure S1 — Phylogenetic (A) and topology (B) trees of initial forty strains. The phylogenetic relationships are based on a total of 13,614 SNPs with a CI = 0.97. Numbers next to branches are bootstrap values of 100 replicates. Branch location of SNP assay targets for identifying spa t003 and t008 are indicated with a red triangle. [file 1471-2334-13-339-S7.tiff]
